# Supplementary material for: Low-Rank Representation of Head Impact Kinematics: A Data-Driven Emulator
Source: Front Bioeng Biotechnol. 2020 Sep 25;8:555493. doi: 10.3389/fbioe.2020.555493 (PMC7546353; doi:10.3389/fbioe.2020.555493)
Supplement: Supplementary file 1 [file Data_Sheet_1.pdf]

Supplementary Materials

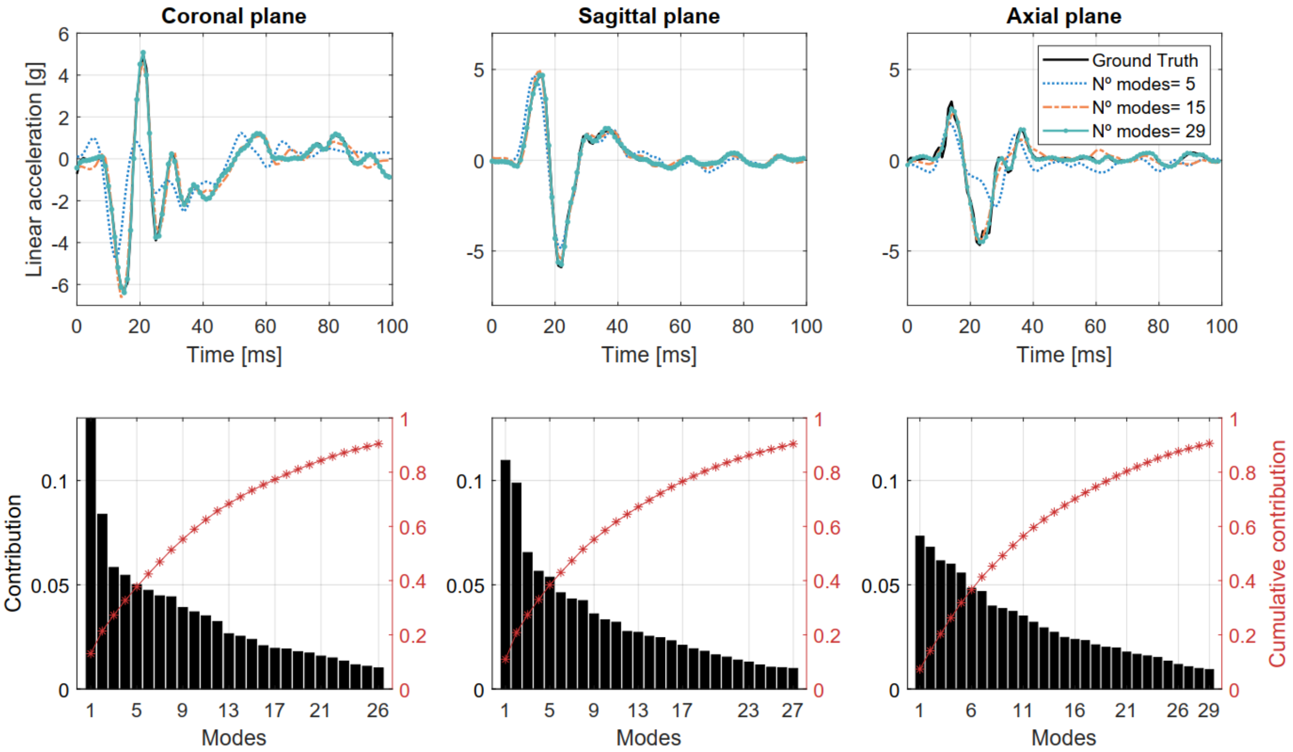

Figure SM.1: *Top:* Representative reconstruction of the linear acceleration data for a single head impact. *Bottom:* Individual and cumulative contribution of the temporal modes derived through PCA for angular velocity in each anatomical direction, constituting over 90% of the ground truth measurements.

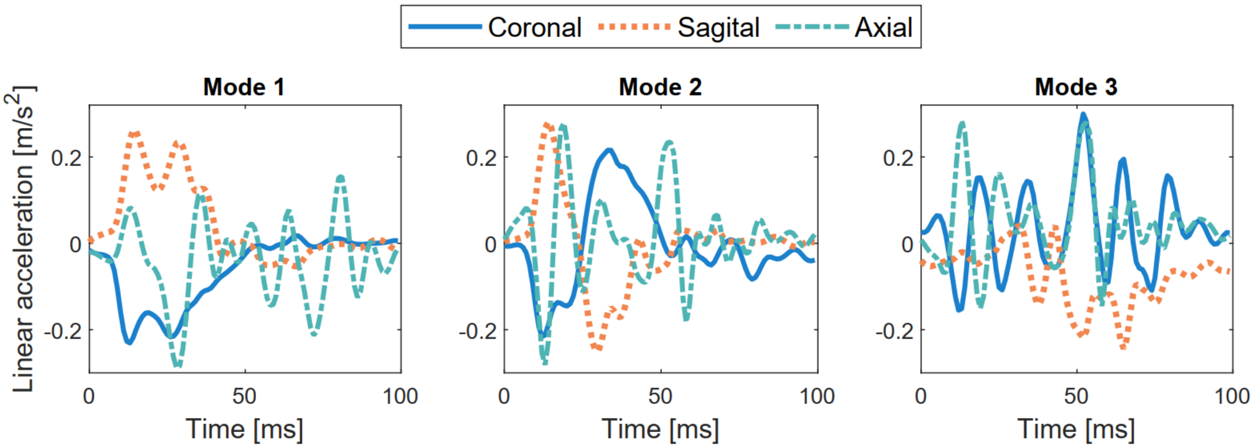

Figure SM.2: The three most relevant temporal modes for linear acceleration for the entire dataset.

Table SM.1: p-values for the different injury metrics obtained by Friedman test.

Parameter	PCA	Tri.	H.S
HIC_{15}	0.7919	< 0.001	< 0.001
RIC_{36}	0.1478	< 0.001	< 0.001
$BrIC$	0.636	0.380	0.311
Coronal BAM	0.955	< 0.001	< 0.001
Sagittal BAM	0.917	0.007	0.001
Axial BAM	0.846	< 0.001	< 0.001
MPS WB	0.731	0.001	< 0.001
MPS CC	0.780	< 0.001	< 0.001
FS CC	0.807	< 0.001	< 0.001

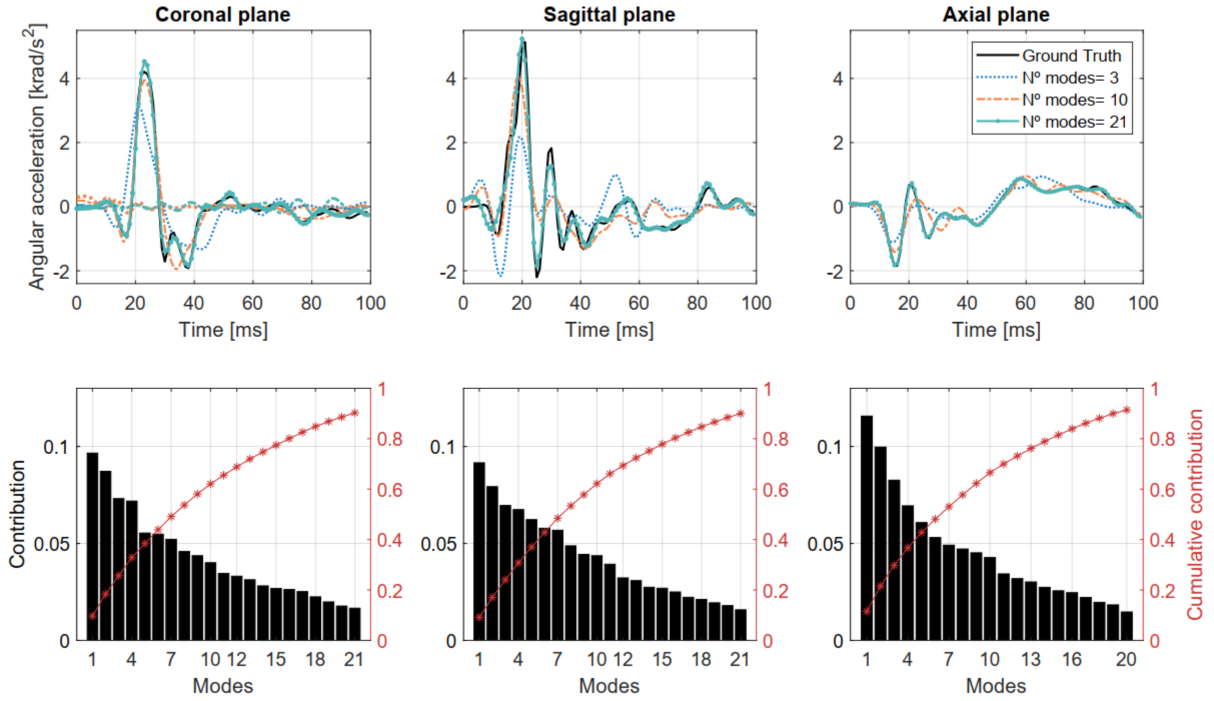

Figure SM.3: *Top:* Representative reconstruction of the angular acceleration data for a single head impact. *Bottom:* Individual and cumulative contribution of the temporal modes derived through PCA for angular velocity in each anatomical direction, constituting over 90% of the ground truth measurements.

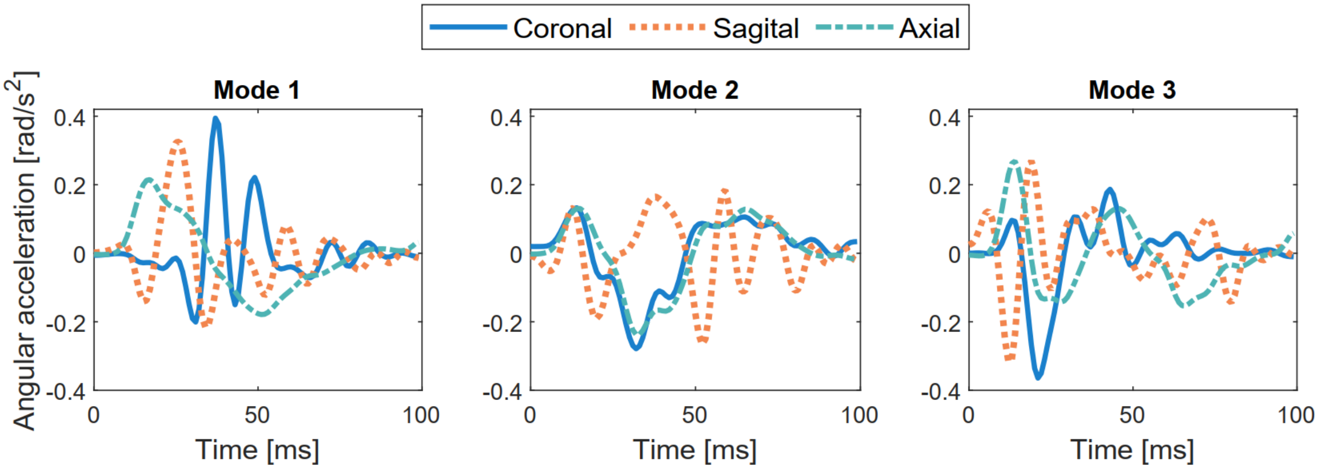

Figure SM.4: The three most relevant temporal modes for angular acceleration for the entire dataset.

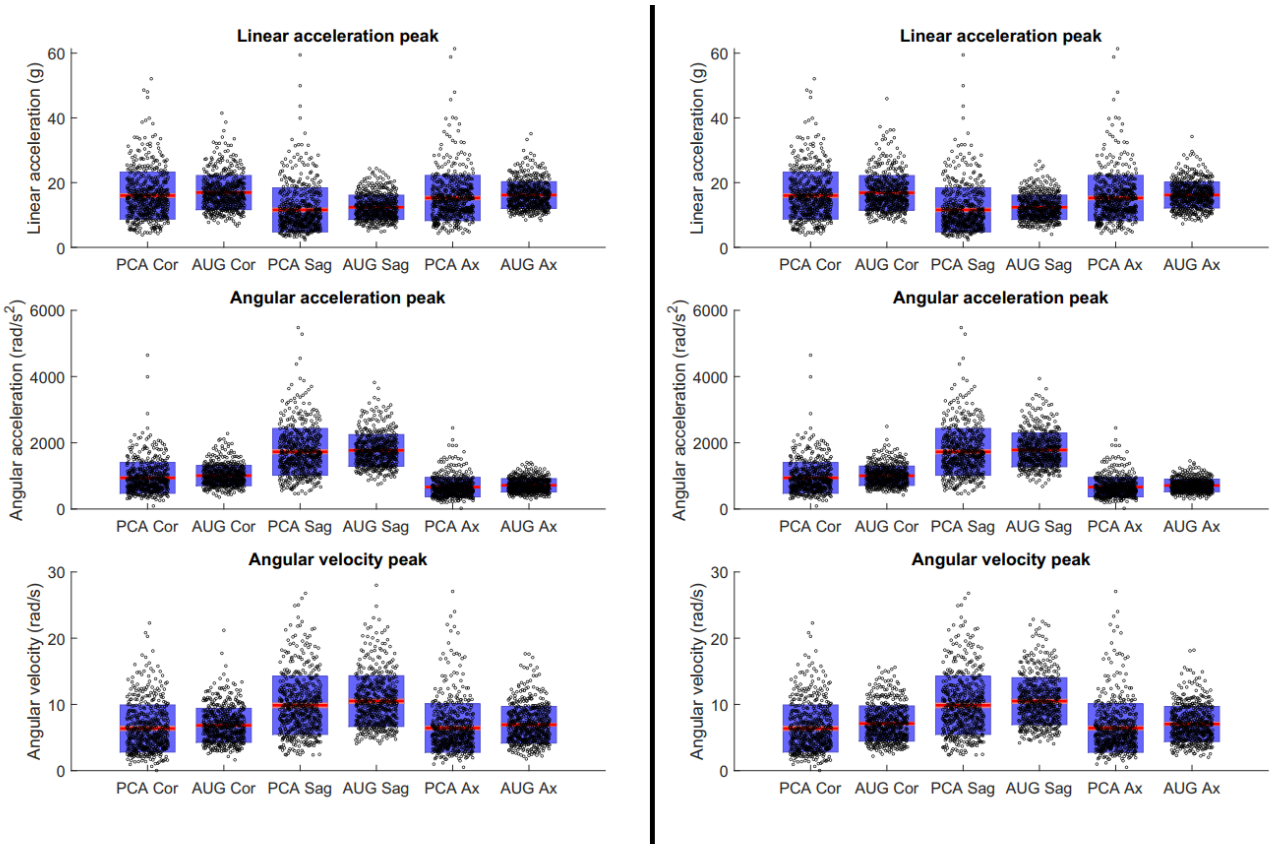

Figure SM.5: Peak distribution comparison between augmented data (two tests) and PCA original data.

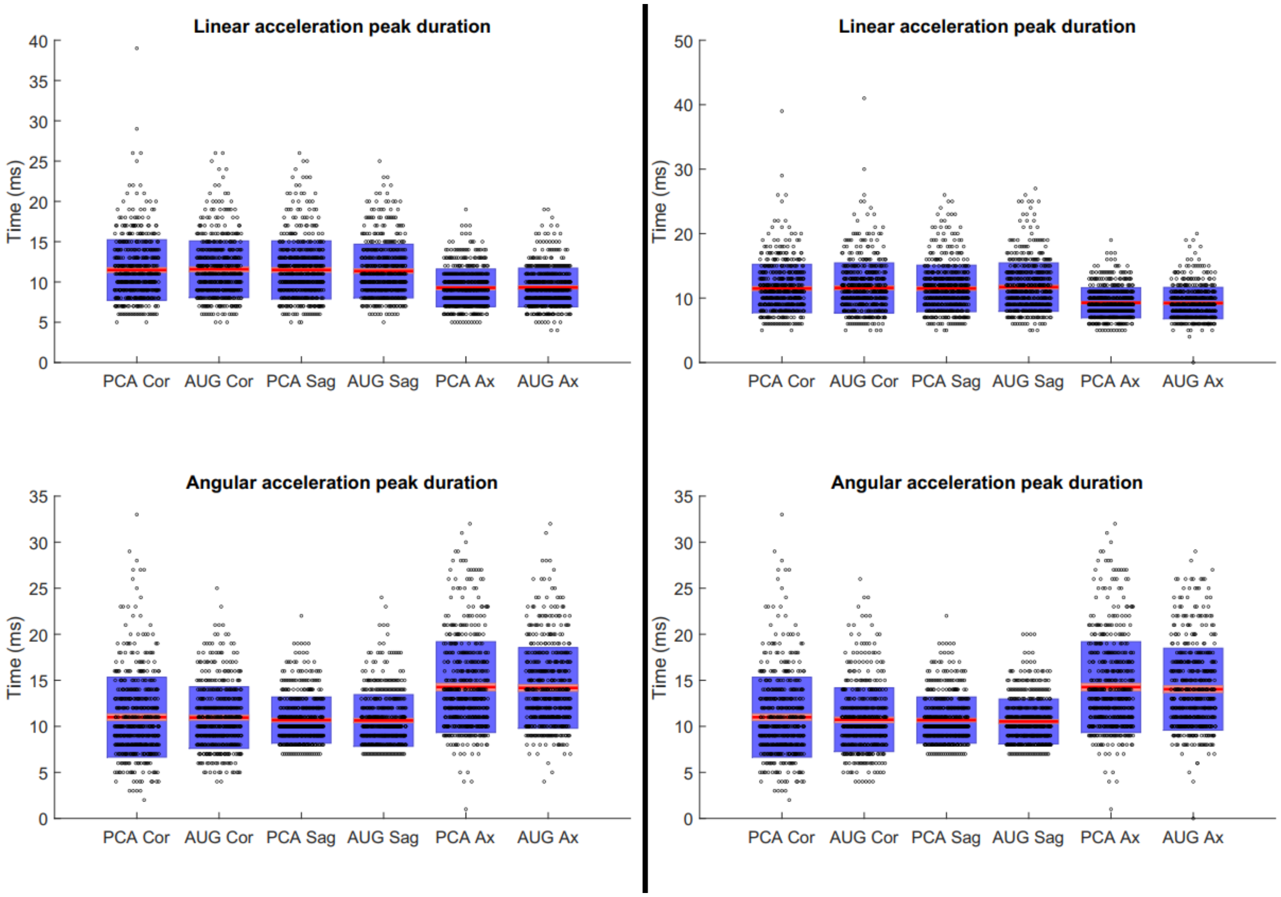

Figure SM.6: Peak duration distribution comparison between augmented data (two tests) and PCA original data.

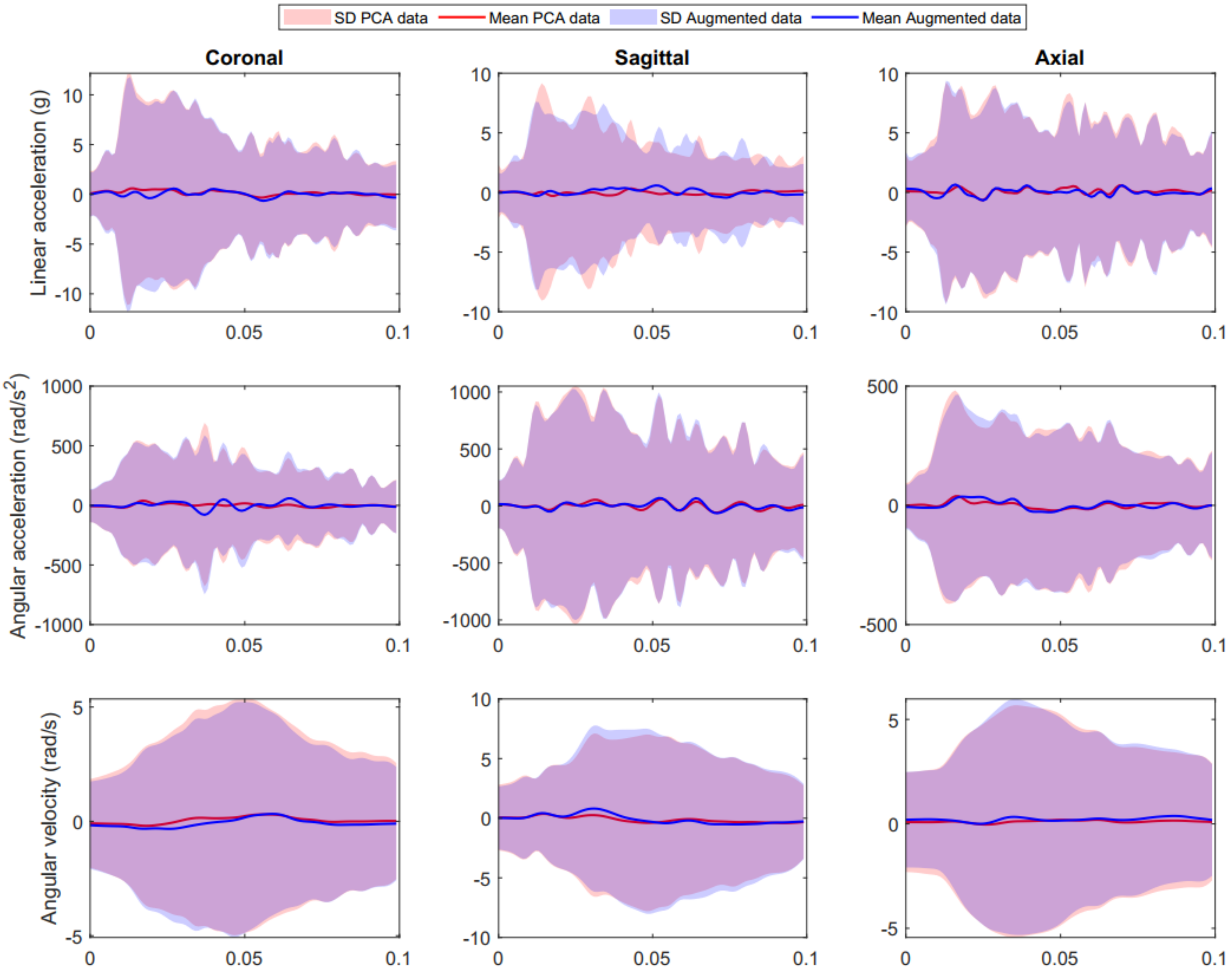

Figure SM.7: Statistical parameters per time point for PCA and an augmented dataset.

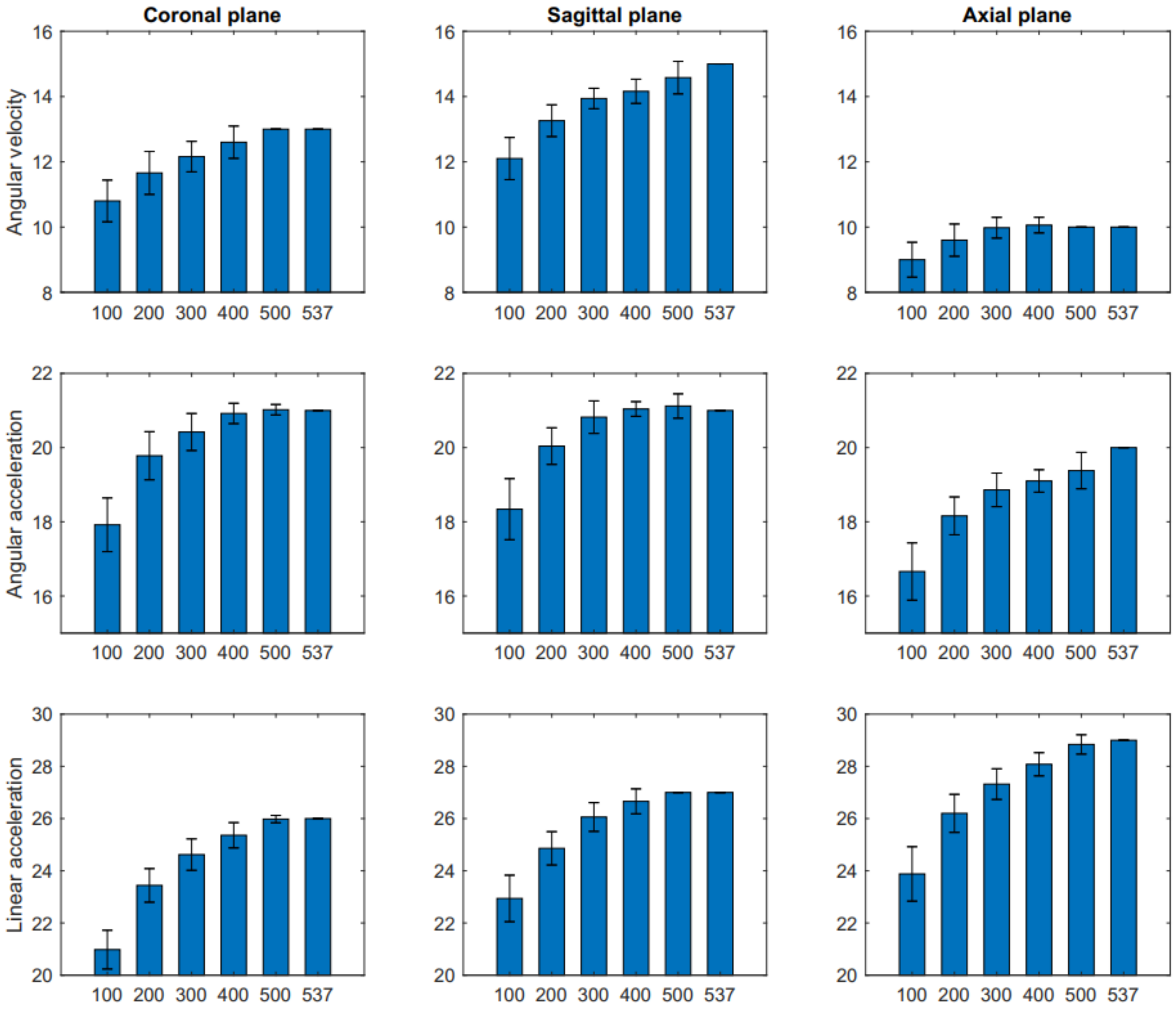

Figure SM.8: Required number of modes to satisfy Equation 1 for random subsets of different size, showing convergence to the values obtained with the entire dataset (537).
